# KONAGAbase: a genomic and transcriptomic database for the diamondback moth, *Plutella xylostella*

**DOI:** 10.1186/1471-2164-14-464

**Published:** 2013-07-09

**Authors:** Akiya Jouraku, Kimiko Yamamoto, Seigo Kuwazaki, Masahiro Urio, Yoshitaka Suetsugu, Junko Narukawa, Kazuhisa Miyamoto, Kanako Kurita, Hiroyuki Kanamori, Yuichi Katayose, Takashi Matsumoto, Hiroaki Noda

**Affiliations:** 1 National Institute of Agrobiological Sciences, Tsukuba 305-8634, Japan

**Keywords:** KONAGAbase, Diamondback moth, *Plutella xylostella*, Insect pest, Genomic and transcriptomic database

## Abstract

**Background:**

The diamondback moth (DBM), *Plutella xylostella*, is one of the most harmful insect pests for crucifer crops worldwide. DBM has rapidly evolved high resistance to most conventional insecticides such as pyrethroids, organophosphates, fipronil, spinosad, *Bacillus thuringiensis*, and diamides. Therefore, it is important to develop genomic and transcriptomic DBM resources for analysis of genes related to insecticide resistance, both to clarify the mechanism of resistance of DBM and to facilitate the development of insecticides with a novel mode of action for more effective and environmentally less harmful insecticide rotation. To contribute to this goal, we developed KONAGAbase, a genomic and transcriptomic database for DBM (KONAGA is the Japanese word for DBM).

**Description:**

KONAGAbase provides (1) transcriptomic sequences of 37,340 ESTs/mRNAs and 147,370 RNA-seq contigs which were clustered and assembled into 84,570 unigenes (30,695 contigs, 50,548 pseudo singletons, and 3,327 singletons); and (2) genomic sequences of 88,530 WGS contigs with 246,244 degenerate contigs and 106,455 singletons from which 6,310 *de novo* identified repeat sequences and 34,890 predicted gene-coding sequences were extracted. The unigenes and predicted gene-coding sequences were clustered and 32,800 representative sequences were extracted as a comprehensive putative gene set. These sequences were annotated with BLAST descriptions, Gene Ontology (GO) terms, and Pfam descriptions, respectively. KONAGAbase contains rich graphical user interface (GUI)-based web interfaces for easy and efficient searching, browsing, and downloading sequences and annotation data. Five useful search interfaces consisting of BLAST search, keyword search, BLAST result-based search, GO tree-based search, and genome browser are provided. KONAGAbase is publicly available from our website (http://dbm.dna.affrc.go.jp/px/) through standard web browsers.

**Conclusions:**

KONAGAbase provides DBM comprehensive transcriptomic and draft genomic sequences with useful annotation information with easy-to-use web interfaces, which helps researchers to efficiently search for target sequences such as insect resistance-related genes. KONAGAbase will be continuously updated and additional genomic/transcriptomic resources and analysis tools will be provided for further efficient analysis of the mechanism of insecticide resistance and the development of effective insecticides with a novel mode of action for DBM.

## Background

The diamondback moth (DBM), *Plutella xylostella*, is one of the most harmful insect pests for crucifer crops worldwide. Control of DBM is made difficult because DBM has rapidly evolved resistance to many classes of conventional insecticides such as pyrethroids, organophosphates, fipronil, spinosad, *Bacillus thuringiensis (Bt)*, and diamides. DBM insecticide resistance is mediated primarily by overexpression of detoxification genes [1-4] and/or gene mutation-derived target insensitivity [5-9]. The life cycle of DBM is very short (approximately 14 days in warm climates), and this is considered as one of the factors for the high insecticide resistance of DBM. Therefore, to efficiently control DBM while suppressing the development of the resistance, it is important to identify and analyze the genes related to the insecticide resistance which will help to (1) clarify the mechanism of DBM insecticide resistance, and (2) to develop new insecticides with a novel mode of action for more effective and environmentally less harmful insecticide rotation.

To accelerate this area of research, a database system which provides genomic and transcriptomic information for DBM is essential. It is important to provide not only sequence data, but also useful annotation information and easy-to-use user interfaces which help researchers to search sequences by homology search, keyword search, and so on through common web browsers. In the Lepidoptera order, to which DBM belongs, the silkworm (*Bombyx mori*) has been used as a representative model insect owing to its well-studied full genome sequence and several useful databases such as KAIKObase [10], SilkDB [11], and SilkBase [12]. Recently, large-scale genome databases of three other lepidopteran insects, MonarchBase for the monarch butterfly (*Danaus plexippus*) [13], Manduca Base for the tobacco hornworm (*Manduca sexta*) [14] and *Heliconius* butterfly genome project website for the Postman butterfly (*Heliconius melpomene*) [15], have been developed based on next generation sequencing. Moreover, lepidopteran EST databases such as SPODOBASE for the fall armyworm (*Spodoptera frugiperda*) [16], WildSilkbase for wild silkmoths [17], and ButterflyBase for various butterflies and moths [18] are all very useful for comparative analysis of lepidopteran insects. Until recently, however, a similar genomic database has not been available for DBM (After public release of KONAGAbase and the submission of this paper, DBM-DB, a genomic database of DBM, was released in late December 2012 [19]).

We describe here the development of KONAGAbase, a genomic and transcriptomic database for DBM which combines comprehensive sequence data with useful annotation information and easy-to-use rich-GUI web interfaces. KONAGAbase is publicly available from our website (http://dbm.dna.affrc.go.jp/px/).

## Construction and content

### EST sequences

Three cDNA libraries were generated from midgut (4th instar larvae from both sexes), egg (both sexes), and testis (4th instar larvae) of the *Bt-*toxin susceptible strain (PXS) of DBMs maintained in our research institute. Total midgut RNA was extracted using RNeasy Mini Kit (Qiagen Inc., USA) according to the manufacturer’s instructions. Midgut cDNA was synthesized using a cDNA Synthesis Kit (TaKaRa Bio Inc., Japan) in accordance with the Gubler–Hoffman method with oligo(dT)_18_ primers and the cDNA was purified by removing small-sized DNA using a CHROMA SPIN + TE-1000 spin columns (Clontech Laboratories Inc., USA). A midgut cDNA library was constructed by cloning cDNA fragments into pBluescript II SK (+) vector (Agilent Technologies Inc., USA) which was then introduced into *Escherichia-coli* HST08 Premium Competent Cells. Egg and testis cDNA libraries were constructed as follows: total RNA was extracted using a QuickPrep Micro mRNA Purification Kit (GE Healthcare UK Ltd, England) according to the manufacturer’s instructions. cDNA was synthesized using SMART RACE cDNA amplification kit (Clontech). First strand cDNA was synthesized using an oligodT primer (5′-TGTGTCTAGAGGATCCGTACCCAGC(T)_30_VN-3′) along with SMART II oligonucleotide (5′-AAGCAGTGGTAACAACGCAGAGTACGCGGG-3′) in the kit. Amplification of cDNA was performed by PrimeSTAR GXL DNA Polymerase (TaKaRa) with SMART technology using the nested universal primer (NUP) (5′-AAGCAGTGGTAACAACGCAGAGT-3′) and 3′-PCR primer (5′-TGTGTCTAGAGGATCCGTACCCAGC-3′) in the kit. An adenine overhang was added to each egg cDNA fragment using Taq polymerase and then the fragments were purified using Sepharose 2B (GE Healthcare). An egg cDNA library was then constructed by cloning the fragments into pGEM-T Easy vector (Promega Inc., USA). Each testis cDNA fragment was amplified using phosphorylated primers and then purified using Sepharose 2B. A testis cDNA library was then constructed by cloning the fragments into Sma I site of pUC18 vector (TakaRa). Each cDNA library was introduced into *E. coli* HST08 Premium Competent Cells.

Raw EST sequences were generated by sequencing the cDNA clones of the abovementioned three cDNA libraries using ABI 3730 XL sequencer. The generated sequences were base-called using the Phred program [20,21] and low quality regions (quality value (QV) < 20) were removed. EST sequences less than 100 bp were discarded. Next, contamination by vector, adapter, linker or primer in the remaining sequences was detected by cross_match program (−minmatch 12 -minscore 20 options used) and BLAST searches against UniVec database file (VecScreen Search Parameters described in [22] were used). Contamination by *E. coli* and/or mitochondria sequences were also detected by blastn (cut-off e-value: 1e-40) with mito and ecoli BLAST databases retrieved from NCBI ftp site [23]. Detected contaminating regions were removed and sequences less than 100 bp in length were discarded. The number of high quality EST sequences generated was 35,618 [12,406 (midgut), 6,904 (egg), and 16,308 (testis)].

EST/mRNA sequences of the DBM (2,033 entries, Nov. 2011) were downloaded from the NCBI nucleotide database [24] and preprocessed using the above described procedure. The number of EST/mRNA sequences added was 1,722, resulting in a total of 37,340 EST/mRNA sequences in our database (Table 1).

**Table 1:** Statistics of the EST/mRNA sequences

**Library**	**No. of sequences**	**GC%**	**Sequence length**			
			**Total**	**Average**	**Median**	**Max**
			**(Kbp)**	**(bp)**	**(bp)**	**(bp)**
midgut	12,406	49.2%	5,960	480.4	508	879
egg	6,904	42.6%	3,082	446.4	453	855
testis	16,308	44.2%	9,691	594.2	633	880
NCBI	1,722	50.1%	1,112	645.6	454	16,113
Total	37,340	45.8%	19,844	531.4	563	16,113

### RNA-seq sequences

Total RNA was extracted from the whole body of the 4th instar DBM larvae (both sexes) of the PXS strain. Preparation of cDNA library from the total RNA and sequencing by Illumina HiSeq 2000 sequencer were performed by Hokkaido System Science Co., Ltd. (Sapporo, Japan). As a result, 201,115,780 single-read (101 bp) RNA-seq sequences (total size = 20,313 Mbp) were generated. RNA-seq sequences were *de novo* assembled by Trinity [25] with ALLPATHS-LG error correction and 147,370 contigs were generated. The GC content was 42.82%. The total, average, median, and maximum lengths were 66.5 Mbp, 451.3 bp, 298 bp, and 11,311 bp, respectively. N50 was 515 bp. The lengths of 9,960 contigs were greater than or equal to 1,000 bp.

### WGS sequences

DNA was extracted from the whole body of the 4th instar DBM larvae (both sexes) of the PXS strain and sequenced using a Roche 454 GS FLX Titanium sequencer. As a result, 5,359,190 raw read sequences were generated. Low quality (QV < 20) regions in the sequences were removed by our custom Perl script implemented based on the modified Mott algorithm of the Phred program. Contaminating sequences were removed using the same procedure used for the ESTs. The number of high quality sequences generated was 5,082,983 totaling 1,925 Mbp, representing approximately 5-fold genomic coverage [estimated genome size of DBM is 370 Mbp (data not shown)]. The GC content was 37.70%. The average and median read lengths were 378.8 bp and 406 bp, respectively.

### Work-flow for constructing sequences in KONAGAbase

**Figure 1:**
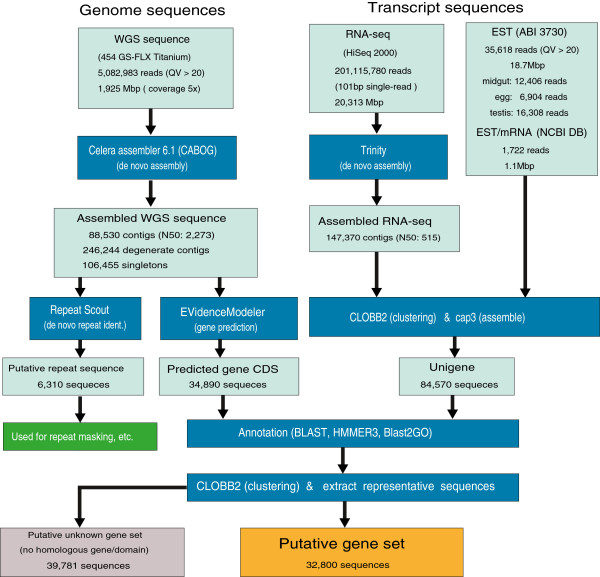
Work-flow for constructing a putative gene set from genomic and transcriptomic sequences in the diamondback moth.

The prepared sequences were processed and analyzed according to the workflow shown in Figure 1. Each process is described below.

### *De novo* assembly of WGS sequences

*De novo* assembly of 5,083,983 high quality WGS read sequences was performed using Celera Assembler version 6.1 with CABOG pipeline [26]. As shown in Table 2, 88,530 contigs (N50: 2,273 bp) were generated with 246,244 degenerate contigs and 106,455 singleton reads which could not be incorporated into contigs. The size of the contigs is approximately 50% of the putative genome size which is almost equal to the sum of the size of degenerate contigs and singleton reads, which may indicate that many of repetitive elements which could not be incorporated into the contigs are contained in the degenerate contigs and singletons. Therefore, we used not only contigs but also degenerate contigs and singleton reads for *de novo* repeat identification and gene prediction (described in later sections) to identify as many repeat sequences and genes as possible.

**Table 2:** Statistics of the WGS assembled sequences

**Sequence type**	**No. of sequences**	**GC%**	**Sequence length**			
			**Total**	**Average**	**Median**	**Max**
			**(Kbp)**	**(bp)**	**(bp)**	**(bp)**
contig	88,530	38.3%	186,028 (51.0%)	2,101.3	1,619	24,960
degenerate contig	246,244	38.8%	147,783 (40.5%)	600.1	558	12,183
singleton	106,455	42.0%	31,160 (8.5%)	292.7	287	727

Percentage values in the “Total” columns are the ratio of the values to the sum of the length of all the assembled sequences. N50 of the contig sequences is 2,273 bp.

### *De novo* identification of repeat sequences

*De novo* identification of putative repeat sequences in the assembled WGS sequences were performed using RepeatScout [27] with “-l 16” (16-mers) option. A total of 6,747 sequences, overlapping by 50 bp or more with WGS contigs, degenerate contigs, or singleton reads, and which appeared 10 times or more, were extracted. These 6,747 sequences were used to query NCBI-nt (non-redundant nucleotide) and NCBI-nr (non-redundant protein) with the blastn/blastx algorithms (at a cut off e-value of 1e-05). The sequences matched with known genes (437; except for repeat sequences such as transposons) were discarded since they may represent non-repeat sequences. The number of putative repeat sequences generated was 6,310. The GC content was 39.8%. The average, median, and maximum lengths were 304 bp, 260 bp, and 1,434 bp, respectively. These sequences were used for repeat masking in the analyses described below.

### Clustering and assembly of transcriptome sequences

To provide a non-redundant, comprehensive transcriptomic DBM dataset, 37,340 ESTs and 147,370 RNA-seq contigs were clustered using CLOBB2 [28] and then assembled using CAP3 [29]. Before performing the clustering, repeat and low complexity regions in the sequences were masked using RepeatMasker [30] with our putative repeat sequences, after which poly-A tails were trimmed using trimest [31]. Sequences of fewer than 100 non-masked bases (7,254 sequences) were rejected. CLOBB was performed with stringent criteria for sequence similarity search (95% or more sequence identity over 50 bp) after which 30,129 clusters, 44,655 pseudo singletons (pseudo singleton consists of a RNA-seq contig) and 1,875 singletons were generated. The clusters were then assembled using CAP3 with 93% overlap identity cutoff, and assembled sequences with transposon-related descriptions (e.g., transposon, transposase, reverse transcriptase, polyprotein, etc.) were identified and discarded based on the result of domain/motif search against Pfam protein database [32] by HMMER3 [33] and homology search against NCBI-nr database by blastx. As a result, 84,570 unigenes consisting of 30,695 contigs, 50,548 pseudo singletons and 3,327 singletons were generated. The GC content was 43.0%. The total, average, median and maximum lengths were 47.8 Mbp, 564.8 bp, 376 bp and 16,249 bp, respectively. The lengths of 10,076 unigenes are greater than or equal to 1,000 bp. CDS prediction was performed using ESTScan [34] and sixpack [31], a total of 84,562 predicted CDSs (more than 15 amino acids) were identified in both the nucleotides and amino acids.

### Gene prediction

Although the generated WGS contigs are relatively small compared with those of model insects such as *B. mori*, those data are still useful for comprehensive preliminary search of target genes. Accordingly, in order to provide useful preliminary information of putative genes, *ab initio* gene prediction was performed against the assembled WGS sequences using AUGUSTUS [35], SNAP [36], and geneid [37] with exonpart/intron hint files (EST/mRNA sequences and RNA-seq contigs of DBM) and with optimized parameter settings for more accurate gene prediction of DBM calculated by us based on the instruction documentation of each software program. Consensus predicted genes were generated by combing results of the three gene prediction programs using EVidenceModeler [38]. Predicted genes with short CDSs (fewer than 200 bp) and/or with transposon-related description identified in the same manner as for the unigenes were discarded. The number of genes generated was 34,890 CDSs of the predicted genes (both the nucleotide CDS and amino acid CDS were generated for each gene). The GC content was 54.8%. The total, average, median and maximum lengths were 16.8 Mbp, 481.1 bp, 360 bp and 11,460 bp, respectively. In the predicted gene CDSs, the number of putative complete genes (including start and stop codons) was 10,682 (30.6%) and the number of putative partial genes was 24,208 (69.4%).

### Generating a putative gene set

To provide a comprehensive putative gene set of DBM, the unigene and predicted genes were clustered by CLOBB2 using criteria identical to those used for the clustering of the unigene, and 72,581 clusters were generated. Of these clusters, 39,781 whose member sequences have no predicted CDSs or no similarity with known domains (Pfam protein database searched by HMMER3) or known proteins (NCBI-nr database and eight gene sets of model insects searched by BLAST as described later) were detected, and 39,781 representative sequences (the longest sequence in each cluster) were selected as a putative unknown gene set in which novel genes might be included. In the remaining clusters, 32,800 representative sequences (Table 3) were selected as a putative gene set based on the criterion in which a sequence with highest BLAST score is preferred (HMMER3 score and sequence length is used in a tie-break). As shown in Table 3, the number of representative sequences derived from unigenes is 20,870 (63.6%) and that derived from predicted gene CDSs is 11,930 (36.4%).

**Table 3:** Statistics of the putative gene set

**Type of representative sequence**	**No. of sequences**	**GC%**	**Sequence length**			
			**Total**	**Average**	**Median**	**Max**
			**(Mbp)**	**(bp)**	**(bp)**	**(bp)**
unigene	20,870	47.3%	18.6	890.3	654	16,249
predicted gene	11,930	54.3%	7.45	624.5	453	11,460
Total	32,800	49.3%	26.0	793.6	570	16,249

### Annotation

The unigene, predicted genes, and the putative gene set were annotated with homology search by BLAST, domain/motif search by HMMER3 (Pfam protein database was used), and GO terms allocated by Blast2GO [39] (the result of the BLAST search for NCBI-nr database was used by Blast2GO). The sequences were compared with various databases for the BLAST search (cutoff e-value: 1e-05), namely, NCBI-nr database, UniRef90 database [40], gene sets (protein sequences) of three lepidopteran insects (*Bombyx mori* (gene set by GLEAN [10,11]), *Manduca sexta* (official gene set (OGS) June 2012 by maker-2.25 [14]), and *Danaus plexippus* (OGS 2.0 [13]) and five other model insects (*Drosophila melanogaster* (gene set of release 5.45 [41])*, Tribolium castaenum* (OGS of Tcas 3.0 assembly [42]), *Anopheles gambiae* (*Anopheles gambiae* PEST annotation, AgamP3.6 [43]), *Apis mellifera* (OGS 1.1 [44]), and *Acyrthosiphon pisum* (OGS 2.1 [45])), and unigenes of two lepidopteran insects (*Spodoptera frugiperda* and *Heliothis virscens*). The two lepidopteran unigene sets, 26,578 unigenes (9,698 contigs and 16,880 singletons) of *S. frugiperda* and 29,094 unigenes (10,355 contigs and 18,739 singletons) of *H. virescens*, were generated by clustering and assembling EST sequences retrieved from SPODOBASE (79,148 ESTs for *S. frugiperda*) [16] and NCBI dbEST [46] (63,504 ESTs for *H. virscens*), respectively, in the aforementioned method used for the unigene of DBM.

## Utility and discussion

### Database system

**Figure 2:**
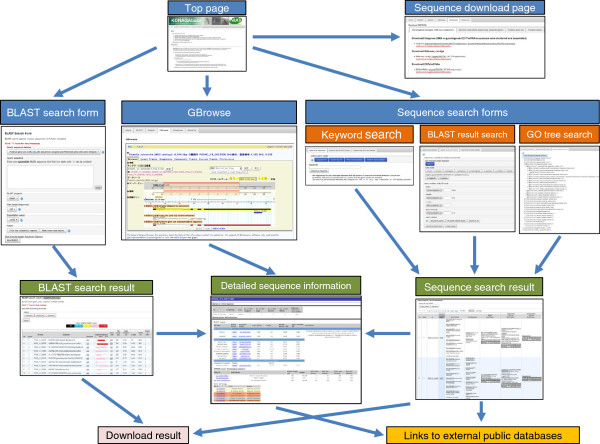
**Feature block diagram of KONAGAbase.** Screenshots of the corresponding web pages are presented for the top page, sequence download page, BLAST search form, GBrowse, BLAST search result, keyword search form, BLAST result search form, GO tree search form, sequence search result, and detailed sequence information page. Sequences searched by BLAST or the three search forms can be downloaded in a tab-delimited text, CSV file, or FASTA file. Links to external public databases include the NCBI database, UniRef90, the Gene Ontology, SPODOBASE, KAIKObase, SilkDB, Manduca Base, MonarchBase, FlyBase, BeetleBase, VectorBase, BeeBase, and AphidBase.

**Figure 3:**
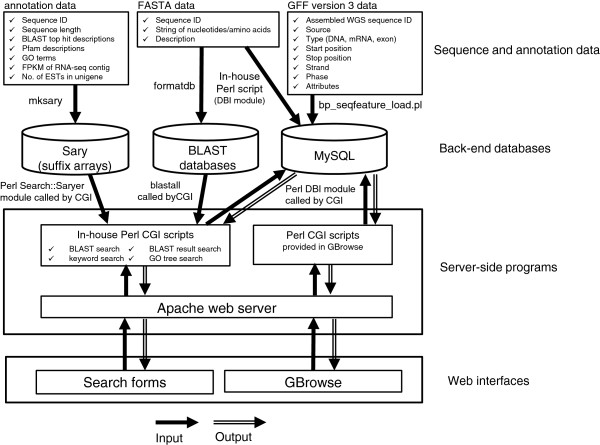
Overview of the database architecture.

We developed a database system for integrating the generated DBM sequences and associated annotation data, and for providing user interfaces to access to the data by standard web browsers. Figure 2 shows the feature block diagram of KONAGAbase. The following web interfaces are provided for efficient and easy searching, browsing, and downloading sequences stored in the database: (1) BLAST search form, (2) keyword search form, (3) BLAST result-based search form, (4) GO tree-based search form, and (5) genome browser powered by GBrowse [47]. All sequences can be downloaded as a zipped FASTA file from a download page. The architecture of the database system consists of three parts: (1) back-end databases, (2) server-side programs, and (3) web interfaces. Figure 3 shows the overview of the database architecture.

### Back-end databases

Generated DBM sequences and associated annotation data were stored using three back-end database software: MySQL (relational database management system), Sary (suffix array library and tools for fast full-text search for huge text files) [48], and BLAST database (generated by formatdb of NCBI BLAST). FASTA format data (sequence ID, string of nucleotides/amino acids, and description based on BLAST top-hit of NCBI-nr) of all DBM sequences were stored into (1) the MySQL database by our in-house Perl scripts using Perl DBI module, and (2) BLAST databases by formatdb. GFF (General Feature Format) version 3 format data required for the GBrowse were also stored into the MySQL database by bp_seqfeature_load.pl provided by BioPerl [49]. The GFF data contains start to end positions of assembled WGS sequences, exons positions and strands of the predicted genes, the unigenes, and the putative gene set on the assembled WGS sequences. Annotation information of each sequence data (tabbed text files containing each sequence ID and corresponding annotation strings) were converted into suffix arrays by mksary provided by Sary.

### Server-side programs

Server-side operations at KONAGAbase are performed in a Linux server (CentOS 6.3). An Apache web server handles queries from web clients through Perl CGI scripts, both generated in-house to perform search functions, and those included in GBrowse. In-house Perl scripts query the suffix arrays using the Perl Search::Saryer module provided by Sary for fast text searches, and send a response to the web interfaces through the web server. Requests for nucleotide and amino acid sequences, as well as for GFF3 data and sequences on GBrowse are handled as queries to a MySQL database via a DBI module.

### Web interfaces

Web interfaces work on client-side standard web browsers and provide users access to KONAGAbase by various search functions. The web interfaces except for GBrowse were implemented by us using HTML, JavaScript, and CSS library with Yahoo! User Interface (YUI) [50] and jQuery [51] which provide Rich-GUI libraries such as tabbed views, sortable tables, tree views, etc., and AJAX (Asynchronous JavaScript and XML) communication libraries for asynchronously sending/receiving data with server-side programs. GBrowse was installed on the Linux server according to the documentation of the software package.

### BLAST search form

A homology search against each type of generated sequence can be performed using the BLAST search form. The result of BLAST search is presented in a rich GUI-based table in a comprehensive summary, and the result (a raw result text and a FASTA file of hit sequence(s)) can be readily selected and downloaded.

### Keyword search form

Each type of sequence can be searched and browsed in the keyword search form. Keywords of “Sequence ID,” “BLAST top hit,” “Pfam ID/Description,” and “GO term ID/Description” can be used in the search form (currently, only keyword search of Sequence ID is available for assembled WGS sequences). Lists of all sequences in each sequence type can be presented if the search is invoked without keywords. Searched sequences are presented in a rich GUI-based summary table with accompanying annotation data. The summary table is sortable by sequence ID, BLAST score, sequence length, and the numbers of member ESTs (only for unigenes) by clicking each title at the top of the table. Each data in the table can be dynamically selected/deselected and easily downloaded as a tab-delimited text, CSV, or FASTA file. For assembled WGS sequences, a link to the GBrowse is available for each sequence in the table. Detailed information of annotated sequences is provided as HTML pages and links to the pages are presented in the summary table. Links to external public databases such as NCBI, UniRef90, the Gene Ontology [52], SPODOBASE, KAIKObase, SilkDB, Manduca Base, MonarchBase, FlyBase [41], BeetleBase [42], VectorBase [43], BeeBase [44], and AphidBase [45] are also presented in the HTML pages.

### BLAST result-based search form

Annotated sequences can be searched based on their annotated BLAST search results using the BLAST result-based search form. Target databases, threshold values of BLAST score (bit-score, identity, e-value, query coverage) can be selected in the form. For example, sequences which are homologous with gene sets of three lepidopteran insects (*B. mori, M. sexta*, and *D. plexippus*) can be searched by selecting the three insects as target databases. Sequences which are not homologous with the three lepidopteran insects can also be searched by additionally selecting the “Search No hit” option. Furthermore, sequences which are homologous with at least one of the three lepidopteran insects (i.e., OR search) can be searched by additionally selecting “OR Search (BLAST result)” option. By selecting threshold values in BLAST score form, for example, highly homologous sequences can also be searched.

### Search by GO term tree viewer

Annotated sequences can also be searched using the GO Tree-based search form in which a graphical tree GO term viewer is provided. Each GO term annotated to the selected type of sequences is presented as a node in the viewer. In each node, the number of sequences annotated with the GO term(s) in the sub-tree of the node is presented. One or more GO terms can be selected using the checkboxes next to each node and sequences annotated with the selected GO terms can be browsed in the summary table. For example, a list of unigenes whose molecular functions are related to transporter activity can be easily searched by checking GO:0005215 (transporter activity) in the sub-tree of molecular function in the GO term viewer and clicking a search button.

### Genome browser powered by GBrowse

GBrowse provides bird's eye and detailed views of the assembled WGS sequences. Three tracks can be selected on the detailed view: (1) predicted gene, (2) unigene, and (3) putative gene set. In each track, exons of each sequence on a selected assembled WGS sequence are visualized. Locations of exons on the genome were determined by EVidenceModeler (for the predicted genes) and exonerate [53] (for the unigenes). A popup balloon is displayed by clicking a sequence on the detailed view, providing links to annotation information page and to detail of exons position page.

## Data analysis

### Homology search result of the putative gene set

Table 4 summarizes the result of a BLAST search for the putative gene set of DBM. Three lepidopteran insects (*B. mori*, *M. sexta*, and *D. plexippus*) in the gene sets show higher similarity (more hits) than NCBI-nr and five other non-lepidopteran insects. Between the three lepidopteran insects, *M. sexta* shows the highest similarity, which is almost equal to that of *D. plexippus*. The unigenes of the two lepidopteran insects (*S. frugiperda* and *H. virscens*) show significantly lower similarity than those of other insects in the gene sets, which may be due to the presence of relatively short and fragmented sequences indicated by lower average query coverage compared with other databases (i.e., there would be many missing genes in the two unigenes). *T. castaenum* shows the highest similarity of the non-lepidopteran insects. In the putative gene set of DBM, 25,344 sequences (77.27%) have similarity with all the three gene sets of the lepidopteran insects. On the other hand, 7,179 sequences (21.89%) of the putative gene set of DBM have no similarity with the five non-lepidopteran insects. Of these 7,179 sequences, 3,088 sequences (9.4%) have similarity with all the three lepidopteran insects, which may indicate that the sequences represent lepidoptera-specific genes. In the putative DBM gene set, 337 sequences have no similarity with all known genes but have one or more homologous domains detected by HMMER3, indicating that these 337 sequences might represent DBM-specific genes.

**Table 4:** Results of BLAST search for the putative gene set

**Database**	**No. of hit (rate of hits)**	**Avg. query coverage**	**Avg. subject coverage**
**Non-redundant protein database**			
NCBI nr	25,290 (77.1%)	72.4%	40.5%
**Gene set of insects**			
*Bombyx mori*	27,659 (84.3%)	69.0%	39.8%
*Manduca sexta*	29,556 (90.1%)	71.0%	36.2%
*Danaus plexippus*	29,518 (90.0%)	72.0%	36.4%
*Drosophila melanogaster*	21,281 (64.9%)	69.2%	34.9%
*Tribolium castaenum*	23,045 (70.3%)	69.6%	35.7%
*Apis mellifera*	21,599 (65.9%)	68.3%	35.7%
*Acyrthosiphon pisum*	21,414 (65.3%)	68.5%	35.8%
*Anopheles gambiae*	21,587 (65.8%)	68.6%	35.5%
**Unigenes of lepidopteran insects**			
*Spodoptera frugiperda*	13,399 (40.9%)	46.4%	40.3%
*Heriothis Virescens*	16,278 (49.6%)	44.9%	38.9%

The average query coverage was calculated by dividing the aligned length by the length of a query sequence (a putative gene of DBM). Similarly, the average subject coverage was calculated by dividing the aligned length by the length of a subject sequence (a sequence of the target database).

### Repeat analysis

The 6,310 putative repeat sequences were classified based on the result of the BLAST search against known repeat sequences: NCBI-nt/nr database, transposable element library of *B. mori* (BmTE library) in KAIKObase (1,960 transposon sequences), and RepBase 16.08 (26,703 repeat sequences) [54]. Homology searches by blastn and tblastx (blastx for NCBI-nr) were performed against each database. The e-value cutoff was 1e-05 and the percent identity cutoffs were 80% for blastn and 30% for blastx/tblastx, respectively. The classification was performed based on the description of the top hit sequences of the BLAST search. As shown in Table 5, 2,399 sequences (38.0%) were classified as transposable elements, and the remaining 3,911 sequences (62.0%) were unclassified, and may contain novel repeat sequences. Approximately 74% of the transposon sequences were classified as non-LTR transposon (LINE/SINE), and the remaining sequences were classified as LTR transposons (20%), DNA transposons (2.5%), and others (3.5%). RepeatMasker was performed with the putative repeat sequences against all the WGS assembled sequences. Therefore, 32.51% of WGS assembly sequences were masked, of which 9.24% were masked by the putative transposon sequences and the remaining 23.27% were masked by the unclassified repeat sequences.

**Table 5:** **Classification of *de novo *identified putative repeat sequences**

**Repeat type**		**No. of putative repeat sequences by Repeat Scout**	**Repeat Masker result**		
			**No. of copies**	**Masked bases (Kbp)**	**Percentage of masked bases (%)**
Transposon	LINE/SINE	1,776	135,174	24,072.6	6.60%
	LTR	482	12,451	2,642.4	0.72%
	DNA	61	15,994	3,825.6	1.05%
	Others	80	28,354	3,187.7	0.87%
Unclassified		3,911	601,647	84,920.6	23.27%
Total		6,310	893,620	118,649.0	32.51%

“Percentage of masked bases” is the percentage of masked bases against the total bases of the WGS contigs, degenerate contigs, and singletons.

### More represented genes among the sequenced transcripts in the midgut, egg, and testis

The top 10 more represented genes among the sequenced transcripts in three tissues (midgut, egg, and testis) were extracted by counting the number of EST sequences contained in the unigene sequences. The genes of the unigenes were identified based on the annotated description of the proteins (NCBI-nr) by BLAST search and on the description of the Pfam domain using HMMER3 search.

In the midgut, as shown in Table 6, genes encoding digestive enzymes (serin proteases, lipases, and carboxypeptidases) are the most represented. In particular, serine protease genes are more significantly represented in the digestive enzymes (approximately 30% of ESTs in the midgut). In the case of housekeeping genes, cytochrome c oxidase genes, ribosomal protein genes, and ATP synthase genes are more represented in the midgut. As shown in Tables 7 and 8, these three genes are also more represented in the egg and the testis (with the exception of the cytochrome c oxidases in the testis). Glucosinolate sulfatase genes, ranked seventh in expression in the midgut, are very important genes for the crucifier specialist DBM. The enzymes encoded by these genes prevent the formation of toxic products arising from the glucosinolates of crucifier plants, thereby enabling DBM to feed on these plants [55]. In the case of fibroin genes, ranked ninth, 10 genes contain a “Fibroin P25” domain (Pfam ID: PF07294) and one gene contains a “Fibroin light chain (L-fibroin)” domain (Pfam ID: PF05849). All the 11 genes have BLAST hits with at least one of the three lepidopteran insects (*B. mori, D. plexippus, M. sexta*). However, this result may be because of contamination of the silk gland, in which fibroin is known to be expressed, in the midgut. To validate the expression of the fibroin genes in the midgut, we performed RT-PCR on RNAs extracted from carefully collected additional midguts and silk glands of the fourth instar larvae using primers for the 10 genes containing the “Fibroin P25” domain and the single gene with the “Fibroin light chain” domain. Transcripts encoded by the 10 genes containing the “Fibroin P25” domain were detected in the midgut, whereas they were not detected in the silk gland (data not shown). On the other hand, the transcript encoded by the single gene containing the “Fibroin light chain” domain was not detected in the midgut, whereas it was detected in the silk gland (data not shown). It is at least likely, therefore, that fibroin genes containing the “Fibroin P25” domains are indeed expressed in the midgut. Currently we have no biological hypothesis for this result. In future experiments, we will analyze in more detail the expression of the fibroin genes in the midgut. The 11 unigene sequences can be easily searched using the keyword search form of KONAGAbase as follows: (1) perform a keyword search with the word “fibroin”; and (2) sort the result table in descending order of the number of EST sequences by clicking the column title of “ESTs (midgut).”

**Table 6:** Top 10 more represented genes among the sequenced transcripts in the midgut

**No.**	**Gene**	**No. of ESTs**	**No. of unigenes including ESTs**	**Ratio of ESTs in the midgut**
1	serin protease	3,563	265	28.72%
2	lipase	669	99	5.39%
3	cytochrome c oxidase	438	16	3.53%
4	ribosomal protein	380	95	3.06%
5	mucin	314	46	2.53%
6	ATP synthase	205	27	1.65%
7	glucosinolate sulfatase	179	12	1.44%
8	carboxypeptidase	147	26	1.18%
9	fibroin	134	11	1.08%
10	ferritin	108	7	0.87%

**Table 7:** Top 10 more represented genes among the sequenced transcripts in the egg

**No.**	**Gene**	**No. of ESTs**	**No. of unigenes including ESTs**	**Ratio of ESTs in the egg**
1	ribosomal protein	1,009	107	14.61%
2	cytochrome c oxidase	450	10	6.52%
3	ATP synthase	112	37	1.62%
4	heat shock protein	99	36	1.43%
5	actin	94	14	1.36%
6	elongation factor	83	19	1.20%
7	cuticle protein	83	27	1.20%
8	nucleoplasmin-like protein	73	2	1.06%
9	myosin	56	6	0.81%
10	zinc finger protein	51	38	0.74%

**Table 8:** Top 10 more represented genes among the sequenced transcripts in the testis

**No.**	**Gene**	**No. of ESTs**	**No. of unigenes including ESTs**	**Ratio of ESTs in the testis**
1	arylphorin-like hexamerin	638	27	3.91%
2	elongation factor	582	26	3.57%
3	initiation factor	329	45	2.02%
4	heat shock protein	246	49	1.51%
5	zinc finger protein	210	111	1.29%
6	ribosomal protein	169	34	1.04%
7	tubulin	165	39	1.01%
8	protein kinase	139	50	0.85%
9	ATP synthase	138	30	0.85%
10	protein disulfide isomerase	117	13	0.72%

In the egg, as shown in Table 7, seven housekeeping genes (encoding ribosomal proteins, cytochrome c oxidases, ATP synthases, heat shock proteins, actins, elongation factors, and myosins) account for the top six and ninth most represented genes. Cuticular protein genes, ranked seventh, and nucleoplasmin-like protein genes, ranked eighth, are weakly represented in the other two tissues. As shown in Table 8, zinc finger protein genes, ranked tenth, are also more represented in the testis, in which they are ranked fifth.

In the testis, (Table 8), arylphorin-like hexamerin genes are the most represented. In insects, hexamerins are storage proteins which are mainly synthesized by the fat body and serve as sources of amino acids for pupae and adults during metamorphosis [56,57]. In the honey bee (*A. mellifera*), it has been confirmed that the hex 70a gene (a hexamerin classified as an arylphorin) is expressed not only in the fat body but also in the ovary and testis, which may imply previously unknown roles for the gene in the developing gonads of the honey bee [58,59]. In DBM, two arylphorin-like hexamerin genes (*PxAry1* and *PxAry2*) have been identified [60], although the expression of the genes in the testis was not evaluated in these studies. All 27 identified unigenes in the testis (these sequences can also be easily searched by the keyword search form with the word “arylphorin-like hexamerin” and sorting with “ESTs (testis)” column) are highly similar (approximately 70%–100% identities) with one of the two genes. Although we carefully removed cells which were adhered to the surface of the testes prior to RNA extraction, these cells, which we consider to represent the fat body, may have contaminated the testes material. To validate the expression of genes in the testis, we performed RT-PCR on RNAs extracted from carefully collected additional testes and previously collected testes of the fourth instar larvae using primers for the two genes. We found that transcripts encoded by both genes were detected in both RNA samples when cycle number of RT-PCR is 30 (data not shown). However, the two genes were barely detectable in the additionally collected RNAs when the cycle number was reduced to 21, whereas they were detected in previously collected RNAs (data not shown). It is likely therefore that the elevated expression of both genes was due to contamination of the fat body into the testis. In future experiments, we will analyze in more detail the expression of genes in the testis.

### Classification of three insecticide resistance related genes

Cytochrome P450 and Glutathione S-transferase (GST) are important detoxification enzyme genes in insects, and an ATP-Binding Cassette (ABC) transporter is considered to be involved in detoxification [61]. These gene superfamilies in the putative gene set were identified by keyword search and then classified by two classification methods. One method is based on unidirectional best hit (UBH) of blastp search. The other method is phylogenetic tree-based method which were used for the classifications of the three gene superfamilies in *B. mori*[62-65]. In general, the former method requires less computational cost but the latter method is more accurate.

Firstly, putative genes annotated with the corresponding highly conserved domains (p450 domain (Pfam ID: PF00067) for Cytochrome P450, GST N-terminal (GST_N) domain (Pfam ID: PF02798) for GST, and nucleotide-binding domain (NBD) (Pfam ID: PF00005) for ABC transporter) were extracted by performing the keyword search against the putative gene set using the corresponding Pfam ID. GST_N domain-containing putative genes annotated with description of non-GST genes such as elongation factor 1 gamma and ganglioside-induced differentiation-associated-protein were discarded. For each extracted putative gene, an amino acid sequence of the corresponding domain was extracted based on the alignment position information in HMMER result. All-to-all comparisons using blastp search were performed for the extracted domain sequences in each gene superfamily, respectively. The domain sequences with 98% or higher identity and coverage were manually merged for removing redundancy. We denote the set of the extracted domain sequences as DS1. Since the putative gene set of DBM is relatively short and fragmented compared with those of other model insects, for more strict classification, we extracted relatively long domain sequences whose lengths are more than half length of the corresponding Pfam domain (37 aa for GST_N domain; 231 aa for p450 domain; 59 aa for NBD) from the DS1. We denote the set of the relatively long domain sequences as DS2. Table 9 summarizes the numbers of genes containing the domain sequences in the DS1 and the DS2, respectively. We speculate that the exact number of genes in the putative gene set may lie between the number of genes in the DS1 and the DS2.

**Table 9:** Number of the putative genes containing highly conserved domains of P450, GST, and ABC transporter genes in DBM

**Extracted set of domain sequences**	**No. of the putative genes (domains) in DBM**		
	**P450 (p450 domain)**	**GST (GST_N domain)**	**ABC transporter (NBD)**
DS1	137 (137)	22 (22)	70 (74)
DS2	61 (61)	20 (20)	54 (57)

Two nucleotide-binding domains (NBDs) can be extracted from one ABC gene. Such ABC genes are included in the DS1 (4 genes) and the DS2 (3 genes).

UBH based classification was performed for the DS1 and the DS2 by performing blastp search (cutoff e-value: 1e-05) against corresponding known genes in insects (1,602 P450 genes retrieved from Cytochrome P450 Homepage [66], 231 GST genes and 2,890 ABC transporter genes retrieved from NCBI database), respectively. The family/class of each DBM gene was identified based on the family/class description in the BLAST top hit. Phylogenetic tree-based classification was performed for the DS2 with the corresponding domain sequences in *B. mori* (23 GST_N domains in 23 GST genes [62]; 79 p450 domains in 84 P450 genes [63]; 70 NBDs in 53 ABC genes [65]) which were extracted in the same manner for the DS2. For the classification of P450 genes, four p450 domain sequences of other insects (CYP321A1 in *Helicoverpa zea*, CYP6BD6 in *Manduca sexta*, CYP347A1 in *Tribolium castaenum*, and CYP304F2 in *Zygaena filipendulae*[66]) were also used. To build a phylogenetic tree of each gene superfamily, amino acid sequences of each gene superfamily were aligned using ClustalW [67] and neighbor-joining method in MEGA5 [68] was performed with a bootstrap of 1,000 replicates, Poisson model for amino acid substitution, and pairwise deletion of gaps. The family/class of each DBM gene was identified based on the family/class descriptions of genes located in the same subtree.

The classification results are shown in Table 10 (P450), Table 11 (GST), and Table 12 (ABC transporter). We denote the classification results by the UBH based method as DS1-UBH and DS2-UBH, and that by phylogenetic tree-based method as DS2-PTB. Generated phylogenetic trees of P450, GST, and ABC transporter genes are shown in Figure S1-S3 (Additional files 1, 2 and 3), respectively. For P450 genes, phylogenetic subtrees of each clan are shown in Figures S4-S7 (Additional files 4, 5, 6 and 7), respectively.

**Table 10:** Number of putative P450 genes in each P450 clan/family

**P450 clan**	**P450 family**	**No. of putative P450 genes in DBM**			**No. of P450 genes in *Bombyx mori***
		**DS1-UBH**	**DS2-UBH**	**DS2-PTB**	
CYP2 clan	CYP15	1	1	1	1
	CYP18	2	2	2	2
	CYP303	2	1	1	1
	CYP304	1	1	1	0
	CYP305	1	1	1	1
	CYP306	1	1	1	1
	CYP307	1	1	1	1
Total		9	8	8	7
CYP3 clan	CYP6	31	21	21	16
	CYP9	14	3	3	7
	CYP321	2	2	2	0
	CYP324	0	0	0	1
	CYP332	0	0	0	1
	CYP337	2	1	1	2
	CYP338	2	0	0	1
	CYP347	1	1	1	0
	CYP354	3	1	1	1
	CYP365	1	1	1	1
Total		56	30	30	30
CYP4 clan	CYP4	20	9	7	12
	CYP340	20	5	5	13
	CYP341	6	0	0	8
	CYP366	4	1	1	1
	CYP367	4	0	2	2
Total		54	15	15	36
Mito. clan	CYP49	3	1	1	2
	CYP301	1	0	0	1
	CYP302	2	2	2	1
	CYP314	2	1	1	1
	CYP315	1	1	1	1
	CYP333	7	3	3	4
	CYP339	2	0	0	1
Total		18	8	8	11
Total (All)		137	61	61	84

The number of putative P450 genes in each P450 clan/family was estimated using two sets of p450 domain (Pfam ID: PF00067) sequences (DS1 and DS2). UBH based classification was performed for DS1 (DS1-UBH) and DS2 (DS2-UBH), respectively. Phylogenetic tree-based classification was performed for DS2 (DS2-PTB). The number of P450 genes in *Bombyx mori* was obtained from [63].

**Table 11:** Number of putative GST genes in each GST class

**GSTclass**	**No. of putative GST genes in DBM**			**No. of GST genes in *Bombyx mori***
	**DS1-UBH**	**DS2-UBH**	**DS2-PTB**	
Delta	4	4	4	4
Epsilon	6	5	6	8
Omega	4	3	3	4
Sigma	2	2	2	2
Theta	3	3	1	1
Zeta	2	2	2	2
Unclassified	1	1	2	2
Total	22	20	20	23

The number of putative GST genes in each GST class in DBM was estimated using two sets of GST N-terminal domain (Pfam ID: PF02798) sequences (DS1 and DS2). UBH based classification was performed for DS1 (DS1-UBH) and DS2 (DS2-UBH), respectively. Phylogenetic tree-based classification was performed for DS2 (DS2-PTB). The number of GST genes in *Bombyx mori* was obtained from [62].

**Table 12:** Number of putative ABC transporter genes in each ABC transporter family

**ABC transporter family**	**No. of putative ABC transporter genes in DBM**			**No. of ABC transporter genes in *Bombyx mori***
	**DS1-UBH**	**DS2-UBH**	**DS2-PTB**	
ABCA	8	7	7	9
ABCB	18	11	11	9
ABCC	20	15	15	15
ABCD	2	2	2	2
ABCE	1	1	1	1
ABCF	3	3	3	3
ABCG	15	12	12	12
ABCH	3	3	3	2
Total	70	54	54	53

The number of putative ABC transporter genes in each ABC transporter family in DBM was estimated using two sets of nucleotide-binding domain (Pfam ID: PF00005) sequences (DS1 and DS2). UBH based classification was performed for DS1 (DS1-UBH) and DS2 (DS2-UBH), respectively. Phylogenetic tree-based classification was performed for DS2 (DS2-PTB). The number of ABC transporter genes in *Bombyx mori* was obtained from [65].

In the case of P450 genes, as shown in Table 10, the numbers of putative genes are very different between the DS1 (137 genes) and the DS2 (61 genes). Especially, there are large difference in P450 families in CYP3 clan (26 more genes in the DS1) and CYP4 clan (39 more genes in the DS1), which may be due to many fragmented genes in the DS1. On the other hand, between two different classification methods for the DS2 (DS2-UBH and DS2-PTB), the numbers of genes classified in each clan are equal, and only two genes in two P450 families (CYP4 and CYP367 families in CYP4 clan) are differently classified. The number of P450 genes in the putative gene set is less than that of *B. mori* (84 genes in 26 families [63]) when compared with that in the DS2, but greater than that in the DS1. In total, the putative gene set covers up to 27 P450 families, which includes 24 families identified in *B. mori*, which may indicate high gene coverage of the putative gene set.

In the case of GST genes, as shown in Table 11, the numbers of putative genes are almost the same between the DS1 (22 genes) and the DS2 (20 genes). Genes in Delta, Sigma, and Zeta classes are equally classified between all methods. Although the number of genes in Epsilon, Theta, and unclassified classes are different between the DS2-UBH and the DS2-PTB, only two genes are differently classified in total. The number of GST genes in the putative gene set is less than that of *B. mori* (23 genes [62]), but the difference is small. In total, both the numbers of genes in the DS1 and the DS2 are less than that of *B. mori* only in Epsilon class.

In the case of ABC transporter genes, as shown in Table 12, the numbers of putative genes in the DS1 and the DS2 are 70 and 54, respectively. Especially, the classification results are exactly the same between the DS2-UBH and the DS2-PTB. The number of ABC transporter genes in the putative gene set is more than that of *B. mori* (53 genes [65]). It is likely that DBM has more xenobiotic resistance-associated ABC transporter genes (ABCB, ABCC and ABCG families [61]) than *B. mori*.

In the above results, the differences between the two classification methods for the DS2 are small. Therefore, our classification result for the DS2 may be useful for gene classifications of other insects even when the simple UBH based method is used. In order to evaluate this, classifications of the above three gene superfamilies in two model insects, *B. mori* and *D. melanogaster*, were performed by the UBH based method using our classification result of the DS2-PTB. The domain sequences of each gene superfamily in *D. melanogaster* (37 GST_N domains in 37 GST genes [62], 86 p450 domains in 90 P450 genes [69], and 87 NBDs in 56 ABC transporter genes [70]) were also extracted in the same manner for the DS2. For P450 genes, the genes in common gene families between DBM and each model insect (66 *B. mori* genes and 59 *D. melanogaster* genes) were evaluated. As a result, in *B. mori*, the numbers of classified ABC transporter and GST genes in each family/class by the UBH based method are exactly the same with those of original classifications while the same is true for ABC transporter and P450 genes in *D. melanogaster*. In *B. mori*, only two genes in CYP367 family were wrongly classified as genes in CYP4 and CYP340 families (all the three families belong to CYP4 clan) while nine GST genes in Epsilon class (14 Epsilon genes in original classification) were wrongly classified as genes in Delta class (11 Delta genes in original classification). Thus, it is likely that our classification results for the three gene superfamilies in DBM are useful for the classification in other insects, and may be more useful for that in lepidopteran insects.

## Conclusions

We have constructed KONAGAbase, a genomic and transcriptomic database for DBM. KONAGAbase provides DBM comprehensive transcriptomic and draft genomic sequences with useful annotation information and easy-to-use rich GUI-based web interfaces, which enables researchers to efficiently search target sequences, such as insect resistance-related genes. The database will be continuously updated and additional genomic/transcriptomic resources and analysis tools will be provided for further efficient analysis of the mechanism of insecticide resistant of DBM and the development of effective insecticides based on novel modes of action.

## Availability and requirements

KONAGAbase is available online at http://dbm.dna.affrc.go.jp/px/. All sequences described in this paper can be downloaded from that site. The 35,618 EST sequences (midgut, egg, and testis) and 88,530 WGS contig sequences have been deposited in DDBJ/EMBL/GenBank databases (accession numbers for EST: HX668193 to HX703879 (except for the following 69 accession numbers: HX680621, HX680703, HX680791, HX680829, HX680976, HX681102, HX681191, HX681237, HX681334, HX681357, HX681393, HX681396, HX681477, HX681686, HX681732, HX681940, HX681967, HX682170, HX682187, HX682239, HX682283, HX682472, HX682496, HX682538, HX682618, HX682646, HX682653, HX683074, HX683079, HX683091, HX683109, HX683147, HX683216, HX683314, HX683379, HX683408, HX683418, HX683503, HX683575, HX683584, HX683640, HX683766, HX683769, HX683801, HX683877, HX683942, HX684123, HX684138, HX684160, HX684181, HX684216, HX684549, HX684908, HX684921, HX685117, HX685365, HX685398, HX685529, HX685763, HX685916, HX686063, HX686196, HX686215, HX686246, HX686293, HX686339, HX686659, HX686822, HX687081); WGS contigs: BAGR-01000001 to BAGR01088530). RNA-seq raw sequence data from HiSeq2000 sequencer and WGS raw sequence data from 454 GS FLX Titanium sequencer have been deposited in DDBJ Sequence Read Archive (accession number for RNA-seq raw data: DRA000654; WGS raw data: DRA000655).

## Abbreviations

ABC: ATP-binding cassette; ABI: Applied Biosystems; AJAX: Asynchronous JavaScript and XML; BLAST: Basic local alignment search tool; Bt: *Bacillus thuringiensis*; CDS: Coding sequence; CLOBB: Cluster on the basis of BLAST similarity; CSS: Cascading Style Sheets; CSV: Comma-separated values; CYP: Cytochrome P450; DBM: The diamondback moth; DDBJ: DNA data bank of Japan; EST: Expressed sequence tags; GFF: General Feature Format; GO: Gene ontology; GUI: Graphical user interface; GST: Glutathione S-transferase; LINE: Long interspersed elements; LTR: Long terminal repeat; NCBI: National Center for Biotechnology Information; QV: Quality value; SINE: Short interspersed elements; UBH: Unidirectional best hit; WGS: Whole genome shotgun.

## Competing interests

All the authors declared no competing interests.

## Authors’ contributions

AJ performed bioinformatics analysis, constructed the database system, and wrote the manuscript. KY participated in the conception and coordination of the study. SK, MU, JN performed cDNA library construction, EST sequencing, and total RNA isolation. YS provided overall knowledge and tools related to bioinformatics analysis and construction of the database system. KM provided insect resources. KK, HK, YK, TM performed WGS sequencing. HN performed cDNA library construction, provided overall knowledge related to data analysis, and helped to draft the manuscript. All authors read and approved the final manuscript.

## Supplementary Material

Additional file 1: Figure S1 Phylogenetic tree of DBM, *Bombyx mori* and other insects P450 genes. The amino acid sequences (232 aa or longer) of p450 domain (Pfam ID: PF00067) were extracted (61 domains in DBM; 79 domains in *B. mori*; 1 domain (CYP321A1) in *Helicoverpa zea*; 1 domain (CYP6BD6) in *Manduca sexta*; 1 domain (CYP347A1) in *Tribolium castaenum*; 1 domain (CYP304F2) in *Zygaena filipendulae)* and aligned using ClustalW. Phylogenetic tree was built using neighbor-joining method in MEGA5 with a bootstrap of 1,000 replicates, Poisson model for amino acid substitution, and pairwise deletion of gaps. Bootstrap values resulting from 1,000 replicates are shown at the branch points. Each DBM gene ID (PxCYP-XXXXXX) corresponds to PXGS_V2_XXXXXX in KONAGAbase. Click here for file

Additional file 2: Figure S2 Phylogenetic tree of DBM and *Bombyx mori* GST genes. The amino acid sequences (38 aa or longer) of Glutathione S-transferase, N-terminal (GST_N) domain (Pfam ID: PF02798) were extracted (20 domains in DBM; 23 domains in *B. mori*) and aligned using ClustalW. Phylogenetic tree was built using neighbor-joining method in MEGA5 with a bootstrap of 1,000 replicates, Poisson model for amino acid substitution, and pairwise deletion of gaps. Bootstrap values resulting from 1,000 replicates are shown at the branch points. Each DBM gene ID (PxGST-XXXXXX) corresponds to PXGS_V2_XXXXXX in KONAGAbase. Click here for file

Additional file 3: Figure S3 Phylogenetic tree of DBM and *Bombyx mori* ABC transporter genes. The amino acid sequences (60 aa or longer) of nucleotide-binding domains (NBDs) (Pfam ID: PF00005) were extracted (57 NBDs from 54 ABC transporter genes in DBM; 70 NBDs from 52 ABC transporter genes in *B. mori*) and aligned using ClustalW. To avoid “No common sites” error in MEGA5, 6 NBDs (3 genes) of *B. mori* were removed, resulting in 64 NBDs from 49 ABC transporter genes. Phylogenetic tree was built using neighbor-joining method in MEGA5 with a bootstrap of 1,000 replicates, Poisson model for amino acid substitution, and pairwise deletion of gaps. Bootstrap values resulting from 1,000 replicates are shown at the branch points. The number (1 or 2) in parentheses is added to ID of each ABC transporter gene containing two NBDs. Each DBM gene ID (PxABC-XXXXXX) corresponds to PXGS_V2_XXXXXX in KONAGAbase. Click here for file

Additional file 4: Figure S4 Phylogenetic subtree of DBM, *Bombyx mori* and other insects P450 genes in CYP2 clan. Click here for file

Additional file 5: Figure S5 Phylogenetic subtree of DBM, *Bombyx mori* and other insects P450 genes of CYP3 clan. Click here for file

Additional file 6: Figure S6 Phylogenetic subtree of DBM, *Bombyx mori* and other insects P450 genes of CYP4 clan. Click here for file

Additional file 7: Figure S7 Phylogenetic subtree of DBM, *Bombyx mori* and other insects P450 genes of Mito. clan. Click here for file

## References

[B1] Li X Schuler MA Berenbaum MR Molecular mechanisms of metabolic resistance to synthetic and natural xenobiotics Annu Rev Entomol 2007 52 231 253 10.1146/annurev.ento.51.110104.151104 16925478

[B2] Bautista MAM Tanaka T Miyata T Identification of permethrin-inducible cytochrome P450s from the diamondback moth, *Plutella xylostella* (L.) and the possibility of involvement in permethrin resistance Pesticide Biochemistry and Physiology 2007 87 1 85 93 10.1016/j.pestbp.2006.06.004

[B3] Baek JH Clark JM Lee SH Cross-strain comparison of cypermethrin-induced cytochrome P450 transcription under different induction conditions in diamondback moth Pestic Biochem Phys 2010 96 1 43 50 10.1016/j.pestbp.2009.08.014

[B4] Sonoda S Molecular analysis of pyrethroid resistance conferred by target insensitivity and increased metabolic detoxification in *Plutella xylostella* Pest Manag Sci 2010 66 5 572 575 10.1002/ps.1918 20146259

[B5] Sonoda S Igaki C Ashfaq M Tsumuki H Pyrethroid-resistant diamondback moth expresses alternatively spliced sodium channel transcripts with and without T929I mutation Insect Biochem Mol Biol 2006 36 12 904 910 10.1016/j.ibmb.2006.09.001 17098165

[B6] Sonoda S Igaki C Tsumuki H Alternatively spliced sodium channel transcripts expressed in field strains of the diamondback moth Insect Biochem Mol Biol 2008 38 9 883 890 10.1016/j.ibmb.2008.06.006 18692135

[B7] Baxter SW Chen M Dawson A Zhao JZ Vogel H Shelton AM Heckel DG Jiggins CD Mis-spliced transcripts of nicotinic acetylcholine receptor alpha6 are associated with field evolved spinosad resistance in *Plutella xylostella* (L PLoS Genet 2010 6 1 e1000802 10.1371/journal.pgen.1000802 20062520 PMC2792709

[B8] Rinkevich FD Chen M Shelton AM Scott JG Transcripts of the nicotinic acetylcholine receptor subunit gene *Pxylα6* with premature stop codons are associated with spinosad resistance in diamondback moth, Plutella xylostella Invert Neurosci 2010 10 1 25 33 10.1007/s10158-010-0102-1 20499126

[B9] Baxter SW Badenes-Pérez FR Morrison A Vogel H Crickmore N Kain W Wang P Heckel DG Jiggins CD Parallel evolution of Bacillus thuringiensis toxin resistance in lepidoptera Genetics 2011 189 2 675 679 10.1534/genetics.111.130971 21840855 PMC3189815

[B10] Shimomura M Minami H Suetsugu Y Ohyanagi H Satoh C Antonio B Nagamura Y Kadono-Okuda K Kajiwara H Sezutsu H KAIKObase: an integrated silkworm genome database and data mining tool BMC Genomics 2009 10 486 10.1186/1471-2164-10-486 19843344 PMC2770533

[B11] Duan J Li R Cheng D Fan W Zha X Cheng T Wu Y Wang J Mita K Xiang Z SilkDB v2.0: a platform for silkworm (*Bombyx mori* ) genome biology Nucleic Acids Res 2010 38 Database issue D453 D456 19793867 10.1093/nar/gkp801 PMC2808975

[B12] Mita K Morimyo M Okano K Koike Y Nohata J Kawasaki H Kadono-Okuda K Yamamoto K Suzuki MG Shimada T The construction of an EST database for *Bombyx mori* and its application Proc Natl Acad Sci USA 2003 100 24 14121 14126 10.1073/pnas.2234984100 14614147 PMC283556

[B13] Zhan S Merlin C Boore JL Reppert SM The monarch butterfly genome yields insights into long-distance migration Cell 2011 147 5 1171 1185 10.1016/j.cell.2011.09.052 22118469 PMC3225893

[B14] Agricultural Pest Genomics Resource Databasehttp://www.agripestbase.org/

[B15] Consortium HG Butterfly genome reveals promiscuous exchange of mimicry adaptations among species Nature 2012 487 7405 94 98 22722851 10.1038/nature11041 PMC3398145

[B16] Nègre V Hôtelier T Volkoff AN Gimenez S Cousserans F Mita K Sabau X Rocher J López-Ferber M d'Alençon E SPODOBASE: an EST database for the lepidopteran crop pest Spodoptera BMC Bioinformatics 2006 7 322 10.1186/1471-2105-7-322 16796757 PMC1539033

[B17] Arunkumar KP Tomar A Daimon T Shimada T Nagaraju J WildSilkbase: an EST database of wild silkmoths BMC Genomics 2008 9 338 10.1186/1471-2164-9-338 18637161 PMC2483293

[B18] Papanicolaou A Gebauer-Jung S Blaxter ML Owen McMillan W Jiggins CD ButterflyBase: a platform for lepidopteran genomics Nucleic Acids Res 2008 36 Database issue D582 D587 17933781 10.1093/nar/gkm853 PMC2238913

[B19] You M Yue Z He W Yang X Yang G Xie M Zhan D Baxter SW Vasseur L Gurr GM A heterozygous moth genome provides insights into herbivory and detoxification Nat Genet 2013 45 2 220 225 10.1038/ng.2524 23313953

[B20] Ewing B Hillier L Wendl MC Green P Base-calling of automated sequencer traces using phred, I. Accuracy assessment Genome Res 1998 8 3 175 185 10.1101/gr.8.3.175 9521921

[B21] Ewing B Green P Base-calling of automated sequencer traces using phred, II. Error probabilities Genome Res 1998 8 3 186 194 9521922

[B22] VecScreen http://www.ncbi.nlm.nih.gov/VecScreen/

[B23] NCBI ftp sitehttp://ftp.ncbi.nih.gov/

[B24] NCBI nucleotide databasehttp://www.ncbi.nlm.nih.gov/nuccore

[B25] Grabherr MG Haas BJ Yassour M Levin JZ Thompson DA Amit I Adiconis X Fan L Raychowdhury R Zeng QD Full-length transcriptome assembly from RNA-Seq data without a reference genome Nat Biotechnol 2011 29 7 644 U130 10.1038/nbt.1883 21572440 PMC3571712

[B26] Miller JR Delcher AL Koren S Venter E Walenz BP Brownley A Johnson J Li K Mobarry C Sutton G Aggressive assembly of pyrosequencing reads with mates Bioinformatics 2008 24 24 2818 2824 10.1093/bioinformatics/btn548 18952627 PMC2639302

[B27] Price AL Jones NC Pevzner PA *De novo* identification of repeat families in large genomes Bioinformatics 2005 21 I351 I358 10.1093/bioinformatics/bti1018 15961478

[B28] Parkinson J Guiliano DB Blaxter M Making sense of EST sequences by CLOBBing them BMC Bioinformatics 2002 3 8 10.1186/1471-2105-3-8 12398795 PMC137596

[B29] Huang XQ Madan A CAP3: A DNA sequence assembly program Genome Res 1999 9 9 868 877 10.1101/gr.9.9.868 10508846 PMC310812

[B30] Smit A Hubley R Green P RepeatMasker Open-3.0 http://www.repeatmasker.org/

[B31] Rice P Longden I Bleasby A EMBOSS: The European molecular biology open software suite Trends Genet 2000 16 6 276 277 10.1016/S0168-9525(00)02024-2 10827456

[B32] Punta M Coggill PC Eberhardt RY Mistry J Tate J Boursnell C Pang N Forslund K Ceric G Clements J The Pfam protein families database Nucleic Acids Res 2012 40 Database issue D290 D301 22127870 10.1093/nar/gkr1065 PMC3245129

[B33] Eddy SR Accelerated Profile HMM Searches PLoS Comput Biol 2011 7 10 e1002195 10.1371/journal.pcbi.1002195 22039361 PMC3197634

[B34] Iseli C Jongeneel CV Bucher P ESTScan: a program for detecting, evaluating, and reconstructing potential coding regions in EST sequences Proc Int Conf Intell Syst Mol Biol 1999 138 48 10786296

[B35] Stanke M Diekhans M Baertsch R Haussler D Using native and syntenically mapped cDNA alignments to improve *de novo* gene finding Bioinformatics 2008 24 5 637 644 10.1093/bioinformatics/btn013 18218656

[B36] Korf I Gene finding in novel genomes BMC Bioinformatics 2004 5 59 10.1186/1471-2105-5-59 15144565 PMC421630

[B37] Blanco E Parra G Guigó R Using geneid to identify genes Curr Protoc Bioinformatics 2007 Chapter 4 Unit 4.3 18428791 10.1002/0471250953.bi0403s18

[B38] Haas BJ Salzberg SL Zhu W Pertea M Allen JE Orvis J White O Buell CR Wortman JR Automated eukaryotic gene structure annotation using EVidenceModeler and the Program to Assemble Spliced Alignments Genome Biol 2008 9 1 R7 10.1186/gb-2008-9-1-r7 18190707 PMC2395244

[B39] Conesa A Götz S García-Gómez JM Terol J Talón M Robles M Blast2GO: a universal tool for annotation, visualization and analysis in functional genomics research Bioinformatics 2005 21 18 3674 3676 10.1093/bioinformatics/bti610 16081474

[B40] Consortium U Reorganizing the protein space at the Universal Protein Resource (UniProt) Nucleic Acids Res 2012 40 Database issue D71 D75 22102590 10.1093/nar/gkr981 PMC3245120

[B41] McQuilton P St Pierre SE Thurmond J Consortium F FlyBase 101--the basics of navigating FlyBase Nucleic Acids Res 2012 40 Database issue D706 D714 22127867 10.1093/nar/gkr1030 PMC3245098

[B42] Kim HS Murphy T Xia J Caragea D Park Y Beeman RW Lorenzen MD Butcher S Manak JR Brown SJ BeetleBase in 2010: revisions to provide comprehensive genomic information for Tribolium castaneum Nucleic Acids Res 2010 38 Database issue D437 D442 19820115 10.1093/nar/gkp807 PMC2808946

[B43] Megy K Emrich SJ Lawson D Campbell D Dialynas E Hughes DS Koscielny G Louis C Maccallum RM Redmond SN VectorBase: improvements to a bioinformatics resource for invertebrate vector genomics Nucleic Acids Res 2012 40 Database issue D729 D734 22135296 10.1093/nar/gkr1089 PMC3245112

[B44] Munoz-Torres MC Reese JT Childers CP Bennett AK Sundaram JP Childs KL Anzola JM Milshina N Elsik CG Hymenoptera Genome Database: integrated community resources for insect species of the order Hymenoptera Nucleic Acids Res 2011 39 Database issue D658 D662 21071397 10.1093/nar/gkq1145 PMC3013718

[B45] Legeai F Shigenobu S Gauthier JP Colbourne J Rispe C Collin O Richards S Wilson AC Murphy T Tagu D AphidBase: a centralized bioinformatic resource for annotation of the pea aphid genome Insect Mol Biol 2010 19 Suppl 2 5 12 20482635 10.1111/j.1365-2583.2009.00930.x PMC4372297

[B46] NCBI dbEST http://www.ncbi.nlm.nih.gov/dbEST/

[B47] Donlin MJ Using the Generic Genome Browser (GBrowse) Curr Protoc Bioinformatics 2009 Chapter 9 Unit 9.9 19957275 10.1002/0471250953.bi0909s28

[B48] sary: a suffix array library and toolshttp://sary.sourceforge.net/

[B49] Stajich JE Block D Boulez K Brenner SE Chervitz SA Dagdigian C Fuellen G Gilbert JG Korf I Lapp H The Bioperl toolkit: Perl modules for the life sciences Genome Res 2002 12 10 1611 1618 10.1101/gr.361602 12368254 PMC187536

[B50] The Yahoo! User Interface Library (YUI) http://yuilibrary.com/

[B51] jQueryhttp://jquery.org/

[B52] Ashburner M Ball CA Blake JA Botstein D Butler H Cherry JM Davis AP Dolinski K Dwight SS Eppig JT Gene ontology: tool for the unification of biology, The Gene Ontology Consortium Nat Genet 2000 25 1 25 29 10.1038/75556 10802651 PMC3037419

[B53] Slater GS Birney E Automated generation of heuristics for biological sequence comparison BMC Bioinformatics 2005 6 31 10.1186/1471-2105-6-31 15713233 PMC553969

[B54] Jurka J Kapitonov VV Pavlicek A Klonowski P Kohany O Walichiewicz J Repbase Update, a database of eukaryotic repetitive elements Cytogenet Genome Res 2005 110 1–4 462 467 16093699 10.1159/000084979

[B55] Ratzka A Vogel H Kliebenstein DJ Mitchell-Olds T Kroymann J Disarming the mustard oil bomb Proc Natl Acad Sci USA 2002 99 17 11223 11228 10.1073/pnas.172112899 12161563 PMC123237

[B56] MR K, JK K, RD R, MC VH, R Z Insect hemolymph proteinsvol. 22: Adv Insect Physiol 1990 299 366

[B57] Telfer WH Kunkel JG The function and evolution of insect storage hexamers Annu Rev Entomol 1991 36 205 228 10.1146/annurev.en.36.010191.001225 2006868

[B58] Martins JR Nunes FM Simões ZL Bitondi MM A honeybee storage protein gene, hex 70a, expressed in developing gonads and nutritionally regulated in adult fat body J Insect Physiol 2008 54 5 867 877 10.1016/j.jinsphys.2008.03.009 18472106

[B59] Martins JR Anhezini L Dallacqua RP Simões ZL Bitondi MM A honey bee hexamerin, HEX 70a, is likely to play an intranuclear role in developing and mature ovarioles and testioles PLoS One 2011 6 12 e29006 10.1371/journal.pone.0029006 22205988 PMC3242770

[B60] Ashfaq M Sonoda S Tsumuki H cDNA characterization and expression analysis of two arylphorin-like hexameric protein genes from the diamondback moth, *Plutella xylostella* (L.) Arch Insect Biochem Physiol 2007 64 4 175 185 10.1002/arch.20168 17366599

[B61] Labbé R Caveney S Donly C Genetic analysis of the xenobiotic resistance-associated ABC gene subfamilies of the Lepidoptera Insect Mol Biol 2011 20 2 243 256 10.1111/j.1365-2583.2010.01064.x 21199020

[B62] Yu Q Lu C Li B Fang S Zuo W Dai F Zhang Z Xiang Z Identification, genomic organization and expression pattern of glutathione S-transferase in the silkworm, *Bombyx mori* Insect Biochem Mol Biol 2008 38 12 1158 1164 10.1016/j.ibmb.2008.08.002 19280710

[B63] Ai J Zhu Y Duan J Yu Q Zhang G Wan F Xiang Z-H Genome-wide analysis of cytochrome P450 monooxygenase genes in the silkworm, *Bombyx mori* Gene 2011 480 1–2 42 50 21440608 10.1016/j.gene.2011.03.002

[B64] Liu S Zhou S Tian L Guo E Luan Y Zhang J Li S Genome-wide identification and characterization of ATP-binding cassette transporters in the silkworm, *Bombyx mori* BMC Genomics 2011 12 491 10.1186/1471-2164-12-491 21981826 PMC3224256

[B65] Xie X Cheng T Wang G Duan J Niu W Xia Q Genome-wide analysis of the ATP-binding cassette (ABC) transporter gene family in the silkworm, *Bombyx mori* Mol Biol Rep 2012 39 7 7281 7291 10.1007/s11033-012-1558-3 22311044

[B66] Nelson DR The cytochrome p450 homepage Hum Genomics 2009 4 1 59 65 19951895 10.1186/1479-7364-4-1-59 PMC3500189

[B67] Thompson JD Higgins DG Gibson TJ CLUSTAL W: improving the sensitivity of progressive multiple sequence alignment through sequence weighting, position-specific gap penalties and weight matrix choice Nucleic Acids Res 1994 22 22 4673 4680 10.1093/nar/22.22.4673 7984417 PMC308517

[B68] Tamura K Peterson D Peterson N Stecher G Nei M Kumar S MEGA5: molecular evolutionary genetics analysis using maximum likelihood, evolutionary distance, and maximum parsimony methods Mol Biol Evol 2011 28 10 2731 2739 10.1093/molbev/msr121 21546353 PMC3203626

[B69] The Insect P450 Sitehttp://p450.sophia.inra.fr/

[B70] Dean M Rzhetsky A Allikmets R The human ATP-binding cassette (ABC) transporter superfamily Genome Res 2001 11 7 1156 1166 10.1101/gr.GR-1649R 11435397

