# Supplementary figures and images for: KONAGAbase: a genomic and transcriptomic database for the diamondback moth, Plutella xylostella
Source: BMC Genomics. 2013 Jul 9;14:464. doi: 10.1186/1471-2164-14-464 (PMC3711893; doi:10.1186/1471-2164-14-464)

CYP2 clan

CYP3 clan

Mito. clan

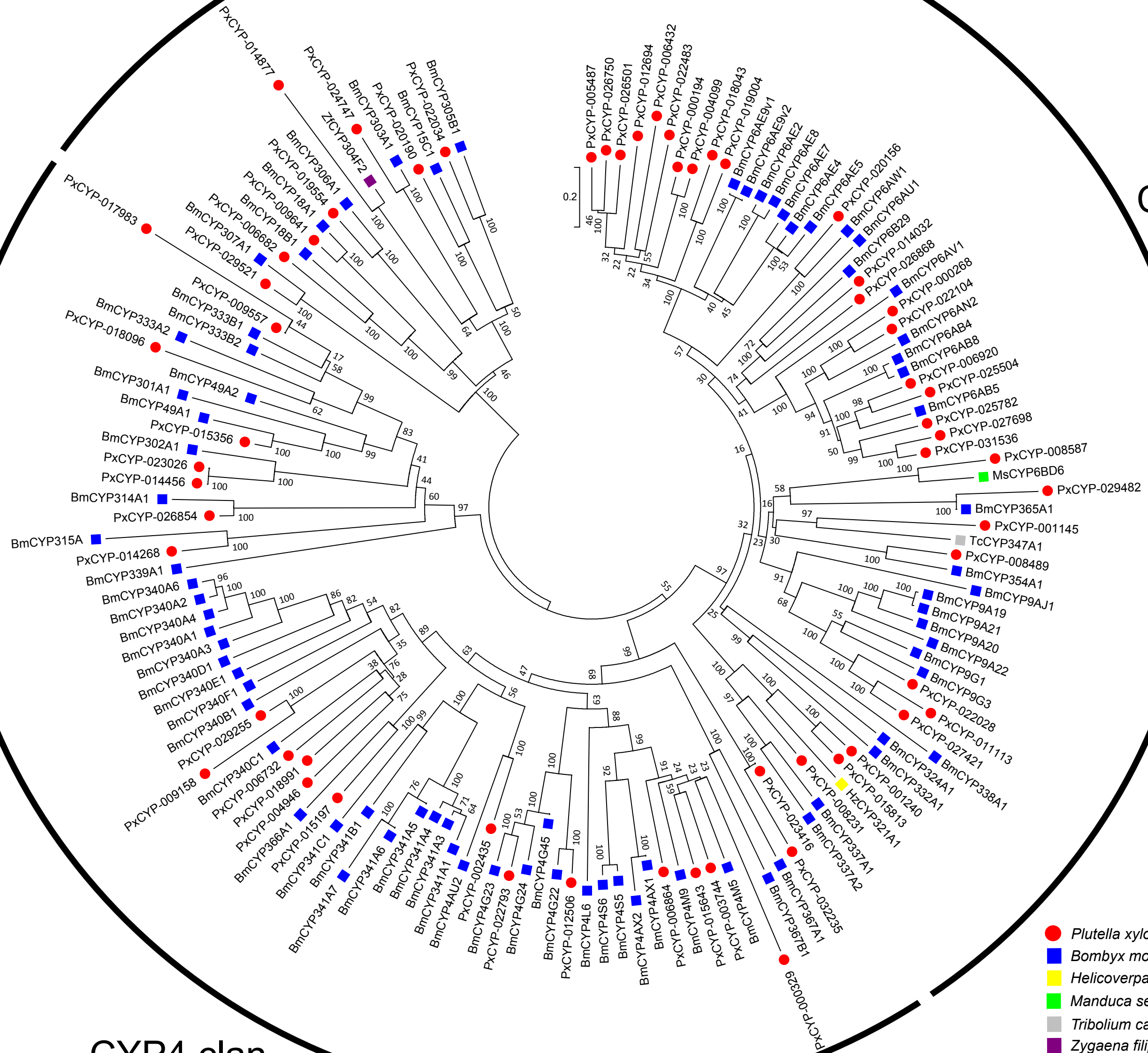

Supplement: Additional file 1: Figure S1 — Phylogenetic tree of DBM, Bombyx mori and other insects P450 genes. The amino acid sequences (232 aa or longer) of p450 domain (Pfam ID: PF00067) were extracted (61 domains in DBM; 79 domains in B. mori; 1 domain (CYP321A1) in Helicoverpa zea; 1 domain (CYP6BD6) in Manduca sexta; 1 domain (CYP347A1) in Tribolium castaenum; 1 domain (CYP304F2) in Zygaena filipendulae) and aligned using ClustalW. Phylogenetic tree was built using neighbor-joining method in MEGA5 with a bootstrap of 1,000 replicates, Poisson model for amino acid substitution, and pairwise deletion of gaps. Bootstrap values resulting from 1,000 replicates are shown at the branch points. Each DBM gene ID (PxCYP-XXXXXX) corresponds to PXGS_V2_XXXXXX in KONAGAbase. [file 1471-2164-14-464-S1.pdf]

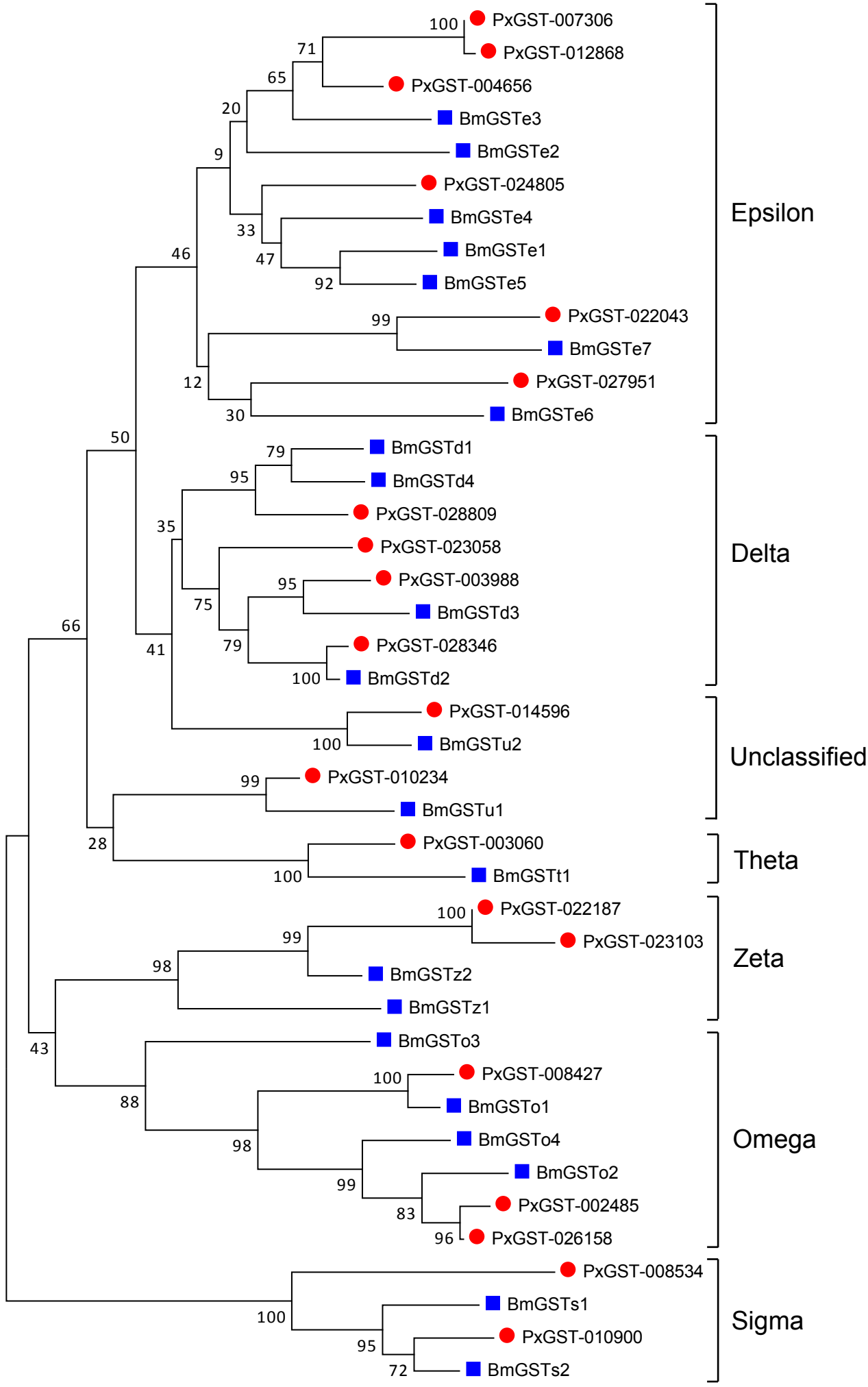

0.2

Supplement: Additional file 2: Figure S2 — Phylogenetic tree of DBM and Bombyx mori GST genes. The amino acid sequences (38 aa or longer) of Glutathione S-transferase, N-terminal (GST_N) domain (Pfam ID: PF02798) were extracted (20 domains in DBM; 23 domains in B. mori) and aligned using ClustalW. Phylogenetic tree was built using neighbor-joining method in MEGA5 with a bootstrap of 1,000 replicates, Poisson model for amino acid substitution, and pairwise deletion of gaps. Bootstrap values resulting from 1,000 replicates are shown at the branch points. Each DBM gene ID (PxGST-XXXXXX) corresponds to PXGS_V2_XXXXXX in KONAGAbase. [file 1471-2164-14-464-S2.pdf]

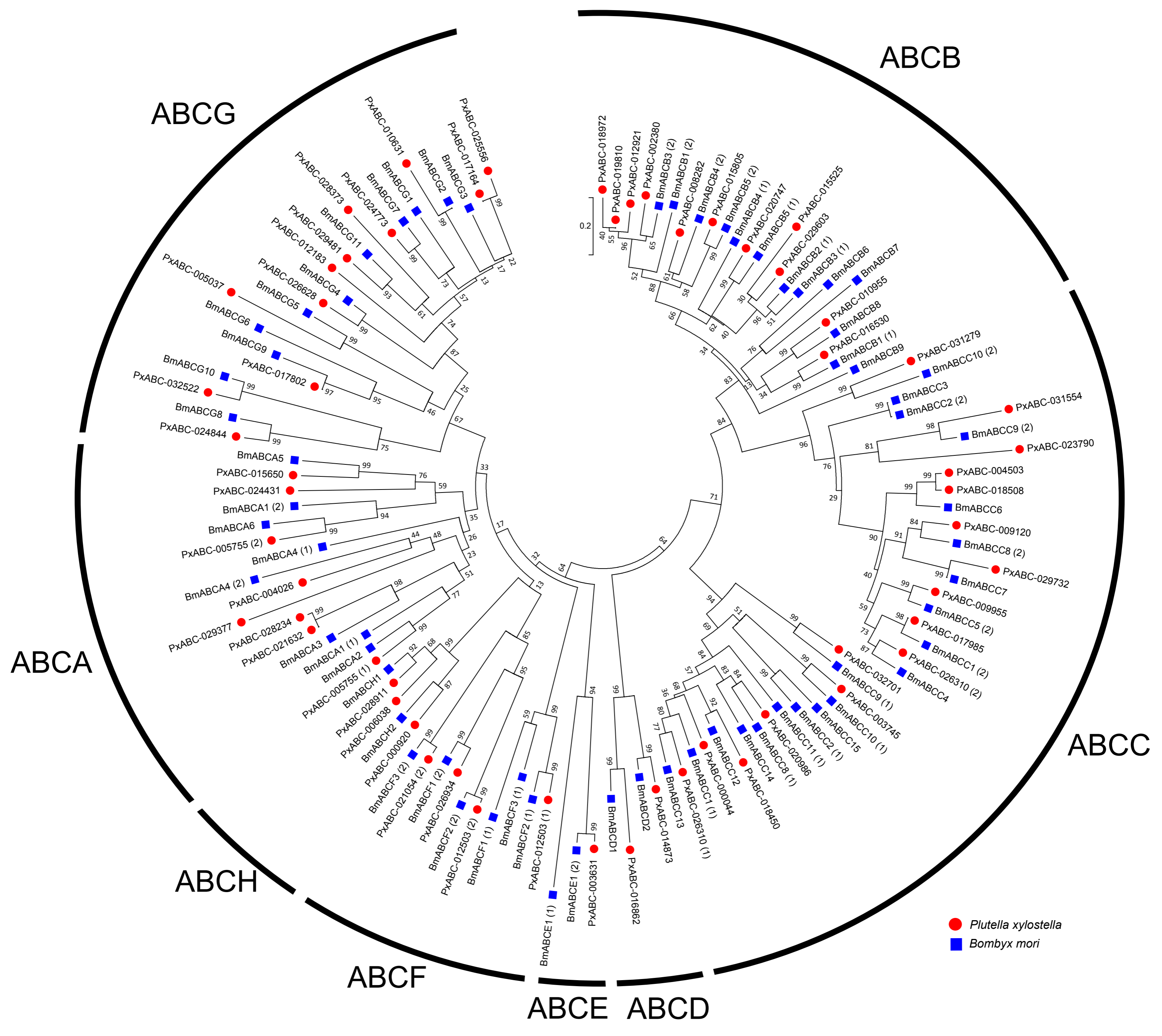

Supplement: Additional file 3: Figure S3 — Phylogenetic tree of DBM and Bombyx mori ABC transporter genes. The amino acid sequences (60 aa or longer) of nucleotide-binding domains (NBDs) (Pfam ID: PF00005) were extracted (57 NBDs from 54 ABC transporter genes in DBM; 70 NBDs from 52 ABC transporter genes in B. mori) and aligned using ClustalW. To avoid “No common sites” error in MEGA5, 6 NBDs (3 genes) of B. mori were removed, resulting in 64 NBDs from 49 ABC transporter genes. Phylogenetic tree was built using neighbor-joining method in MEGA5 with a bootstrap of 1,000 replicates, Poisson model for amino acid substitution, and pairwise deletion of gaps. Bootstrap values resulting from 1,000 replicates are shown at the branch points. The number (1 or 2) in parentheses is added to ID of each ABC transporter gene containing two NBDs. Each DBM gene ID (PxABC-XXXXXX) corresponds to PXGS_V2_XXXXXX in KONAGAbase. [file 1471-2164-14-464-S3.pdf]

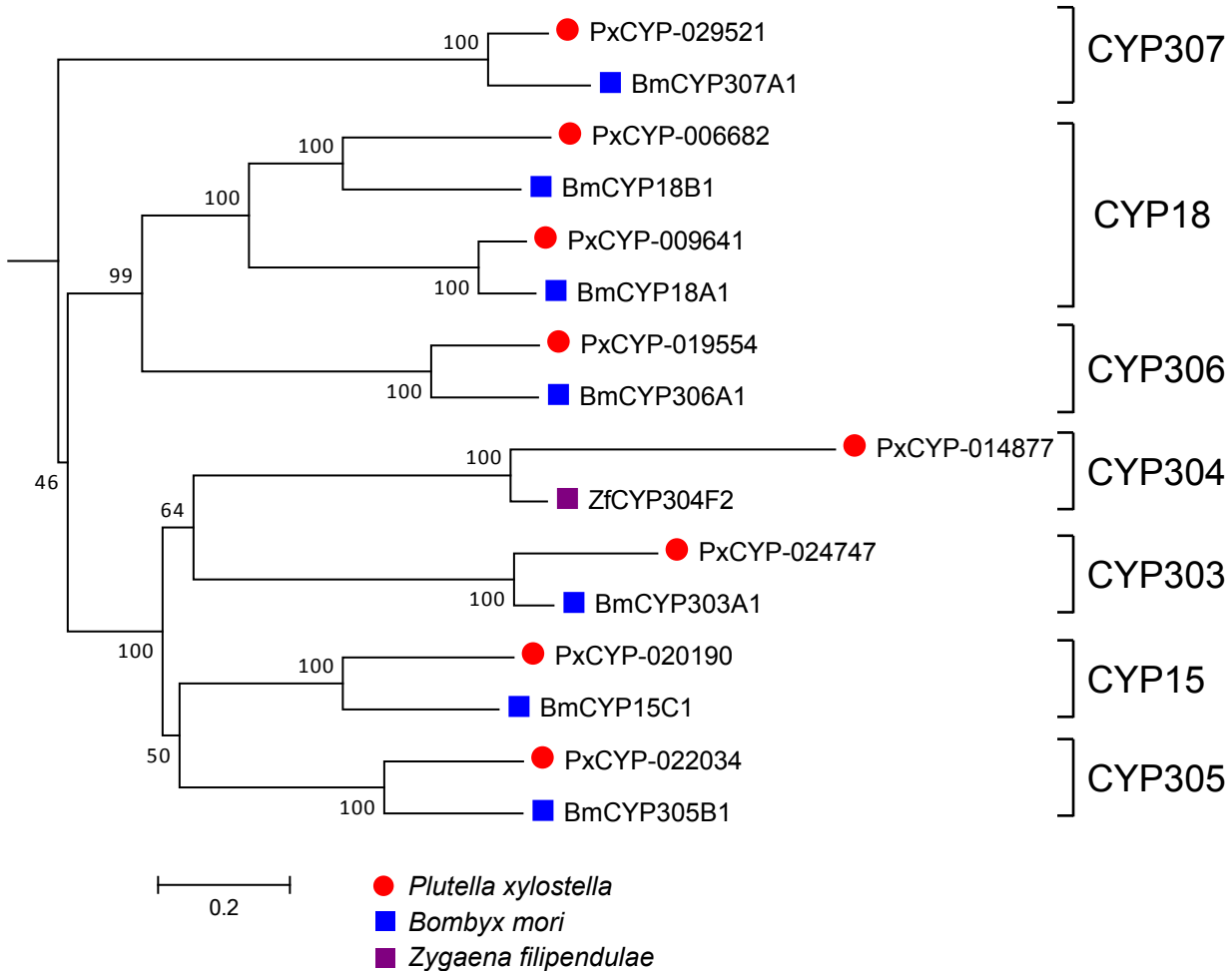

Supplement: Additional file 4: Figure S4 — Phylogenetic subtree of DBM, Bombyx mori and other insects P450 genes in CYP2 clan. [file 1471-2164-14-464-S4.pdf]

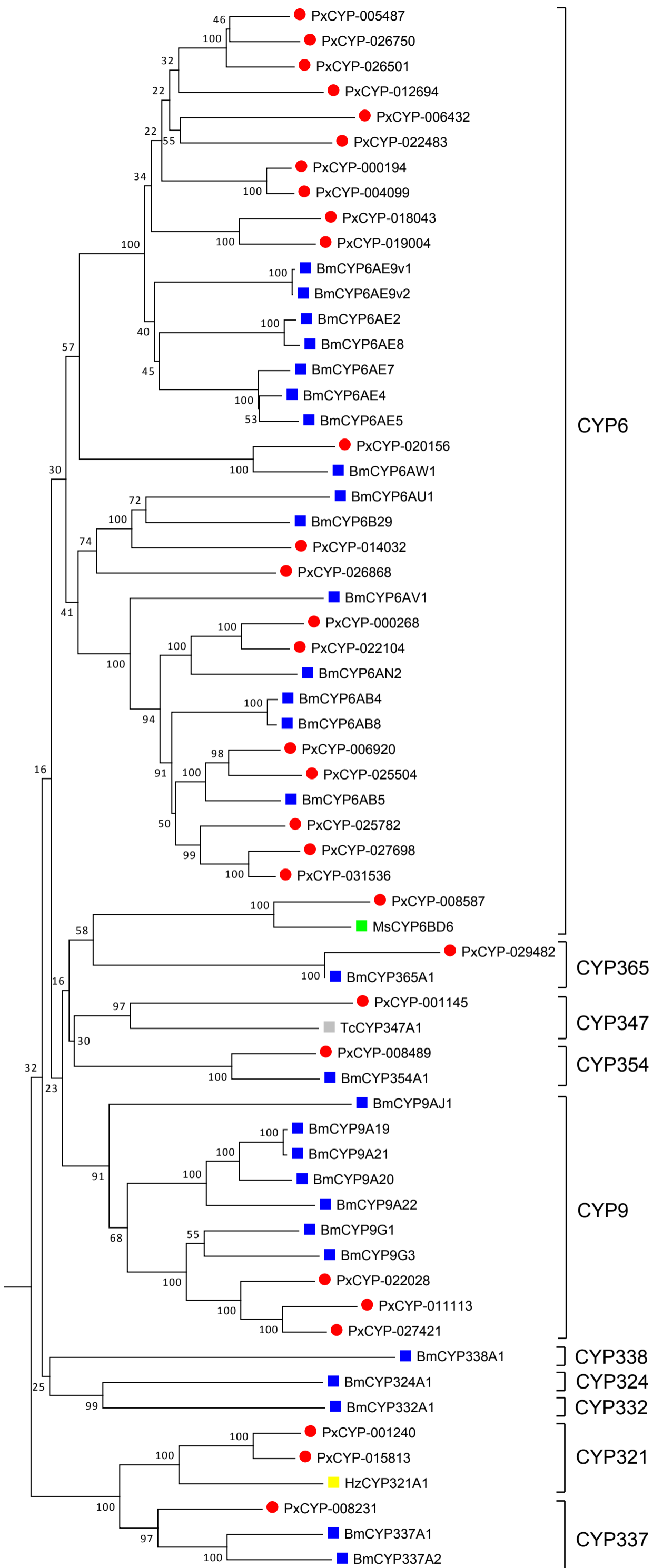

0.1

● *Plutella xylostella* ■ *Manduca sexta*  
■ *Bombyx mori* ■ *Tribolium castaneum*  
■ *Helicoverpa zea*

Supplement: Additional file 5: Figure S5 — Phylogenetic subtree of DBM, Bombyx mori and other insects P450 genes of CYP3 clan. [file 1471-2164-14-464-S5.pdf]

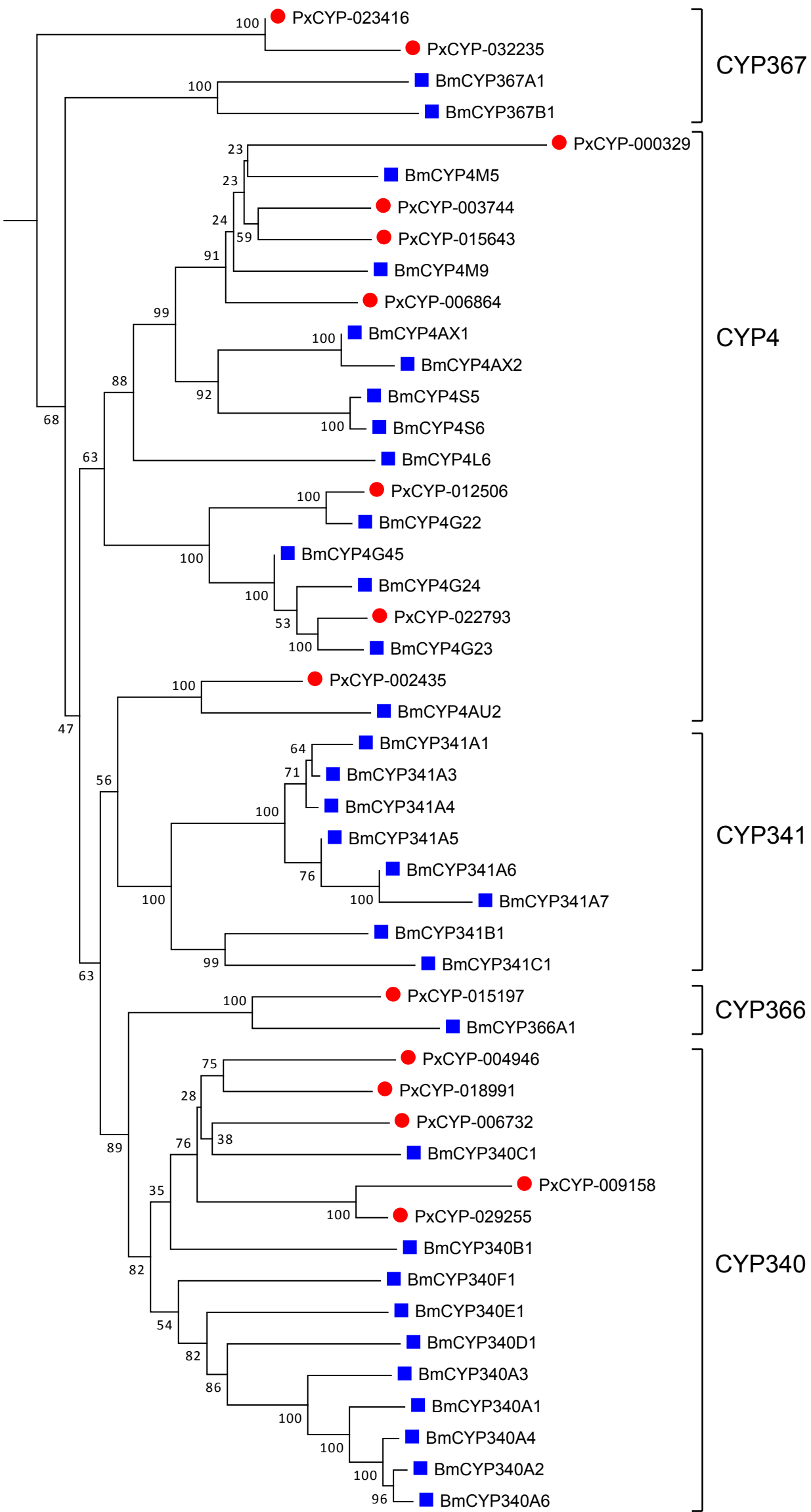

0.1

● *Plutella xylostella*  
■ *Bombyx mori*

Supplement: Additional file 6: Figure S6 — Phylogenetic subtree of DBM, Bombyx mori and other insects P450 genes of CYP4 clan. [file 1471-2164-14-464-S6.pdf]

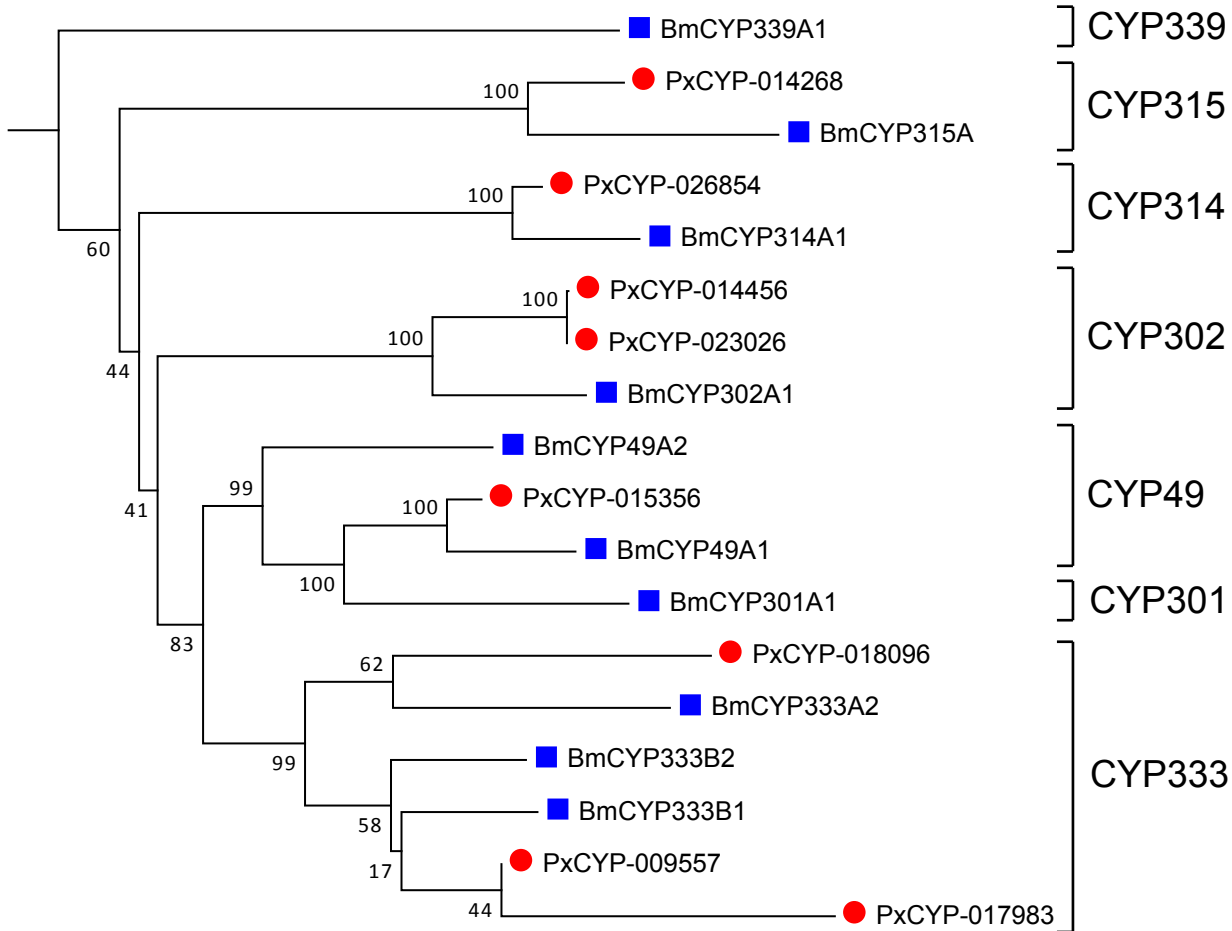

0.2

● *Plutella xylostella*

■ *Bombyx mori*

Supplement: Additional file 7: Figure S7 — Phylogenetic subtree of DBM, Bombyx mori and other insects P450 genes of Mito. clan. [file 1471-2164-14-464-S7.pdf]
